# A Simultaneous and Continuous Excitation Method for High-Speed Electrical Impedance Tomography with Reduced Transients and Noise Sensitivity

**DOI:** 10.3390/s18041013

**Published:** 2018-03-28

**Authors:** Antoine Dupré, Saba Mylvaganam

**Affiliations:** 1 Private Practice, Jouquetti, 05400 Furmeyer, France; 2 Department of Electrical Engineering, IT and Cybernetics, Faculty of Technology, Natural Sciences and Maritime Sciences, University College of Southeast Norway, 3901 Porsgrunn, Norway; saba.mylvaganam@usn.no

**Keywords:** electrical impedance tomography (EIT), high-speed, noise sensitivity, ONe Excitation for Simultaneous High-speed Operation Tomography (ONE-SHOT), projections, simultaneous excitation of electrodes, tomography

## Abstract

This paper presents a concept for soft field tomographic scan of all the projections of electromagnetic waves emanating from an array of electrodes. Instead of the sequential excitation of all pairs of electrodes in the list of all projections, the new method present here consists of a single and continuous excitation. This excitation signal is the linear combination of the excitation signals in the projection set at different AC frequencies. The response to a given projection is discriminated by selecting the corresponding AC frequency component in the signal spectra of the digitally demodulated signals. The main advantage of this method is the suppression of transients after each projection, which is particularly problematic in electrical impedance tomography due to contact impedance phenomena and skin effect. The second benefit over the sequential scan method is the increased number of samples for each measurement for reduced noise sensitivity with digital demodulation. The third benefit is the increased temporal resolution in high-speed applications. The main drawback is the increased number of signal sources required (one per electrode). This paper focuses on electrical impedance tomography, based on earlier work by the authors. An experimental proof-of-concept using a simple 4-electrodes electrical impedance tomographic system is presented using simulations and laboratory data. The method presented here may be extended to other modalities (ultrasonic, microwave, optical, etc.).

## 1. Introduction

Tomography is a probing technique based on imaging using penetrating waves transmitted and received by a pair of transceivers on the boundary of the probed volume. X-ray tomography is the most well established tomography technique, with many medical and industrial applications. Excitation modalities using γ-rays, microwave, ultrasonic, electromagnetic and optical waves are other options for tomography.

Experimentally, in tomography, the system response to a set of projections based on transmitted and received signals needs to be measured. In earlier X-ray tomography concepts, both a single source and detector had to be rotated around the section of the region of investigation (ROI) to be imaged. With the inclusion of arrays of detectors, only the rotational speed of the source and the response time of each detector in the array determined for the frame acquisition rate. Recent X-ray systems for high-speed imaging use a set of X-ray sources that are activated sequentially. 

Electrical impedance tomography (EIT) is another tomographic method. A set of electrodes is positioned on the boundary of the ROI to be imaged. In the standard approach, pairs of electrodes in the projection set are sequentially activated using multiplexers or electronic switching. The voltage measurements on the surrounding electrodes provide information concerning the electrical properties of the ROI. A projection corresponds to the selection of an electrode pair for excitation. The system response to all excitations in the projection set provides a frame of tomographic data that can be used to reconstruct the image of the ROI. In the context of this paper, a projection from the sensor array is one set of data forming the basis for a frame for realizing the tomographic image of the cross-section under study. Different data collection strategies [1] can be used to select the projections in the tomographic set generated in each frame, determining the sensitivity of the tomographic system and the resolution attained in the tomograms. The selection of the drive patterns can be kept flexible to allow for in-situ correction of the sensitivity of the tomograph [2]. Most common projections consider a pair of electrodes, one being connected to the source and the other being connected to the drain of the electrical current supply functioning as the source using the analogy of the X-ray tomographic system.

Electrical tomography techniques feature potentially high frame-data acquisition rate, which is of interest in flow studies involving rapidly evolving flow regimes [3]. Some challenges are specific to the high-speed operation of any type of tomography. Increasing the number of projections (i.e., the spatial resolution) and decreasing the acquisition time (i.e., temporal resolution) lead to degradation of the measurements. Because of the ill-posedness of the inverse problem in soft-field tomography, improvement of the impedance estimations is essential for increasing the image resolution.

In high-speed electrical impedance tomography (EIT) for flow monitoring/imaging of ionic solutions, significant difficulties are caused by the transient voltages resulting from electrode-electrolyte contact impedances. The measurements procedures require dead time and/or complex signal processing techniques or hardware modifications to mitigate this problem [4]. In previous work with high-speed EIT, the authors have observed significant bias in the measurements right after multiplexing to a different voltage excitation configuration [5]. The signals had been sorted in four categories. The first period of measurements involving an electrode recently used for excitation could be biased by 10–100% (relative to settled values). Measurements in the three other categories could be bias by 1–2%. Furthermore, significant difference between electrodes could be observed, probably due to the unavoidable differences of surface state of the electrode-electrolyte interface. The standard approach to reducing transient effects has been to discard the measurements right after multiplexing operation, referred to as dead time. This has long remained a clear limitation in high-speed EIT. An advanced approach has considered hardware modifications to mitigate this problem [4]. However, the dead time would only be decreased. Another approach has consisted in modified signal processing and model based estimation [6].

The concept of Frequency-Division Multiplexing (FDM) consists in differentiating excitation signals in the frequency domain [7,8]. Simultaneously applied excitations at different AC frequencies can be discriminated with demodulation. While this is often discussed in the context of Multi-Frequency EIT, it is seldom discussed in the context of reduction of multiplexing steps in standard EIT. Yet, the perspectives are highly interesting. The consequence of FDM is that the number of samples (i.e., sampling time) available for demodulation is increased. Thereby, the sensitivity to noise of the complex impedance measurements is reduced and the temporal resolution is improved. 

While recent work on multiple excitation capacitance polling [9] opens new prospects for high-speed electrical tomography, it does not tackle the issue posed by effects of transients on measurement accuracy. Authors subdivide the electrodes into excitation and receiving sets. Each excitation electrode is excited with a different frequency. The signals at the receiving electrodes are simultaneously sampled and demodulated using quadrature technique with sine and cosine functions at the frequencies used for excitation. With this technique, the system response can be separated. At the next step of the tomographic sequence, the electrodes will be allocated to a different set yielding a different function. Smart decision can help reduce the number of steps in a tomographic sequence down to three for an 8-electrode sensor, thereby reducing the time needed for frame acquisition. Yet, the transients caused by multiplexing the excitation signal(s) remain in this method, which is a hybrid implementation of FDM.

In an older and neglected conference paper [10], a method that excites simultaneously all electrodes with the same AC frequency but with different binary encoding was suggested. No design for practical implementation was discussed. The authors show that if the binary sequences are chosen to have low cross-correlation but high autocorrelation properties, the signals measured can be separated and the contribution from each electrode can be recovered without ambiguity. The authors do not address the benefit from going toward a single and continuous excitation strategy.

This concept of Frequency-Division Multiplexing fully replacing Time-Division Multiplexing is at the focus of the present study. This enables a continuous excitation strategy and will in principle tackle the issue of transients caused by multiplexing in the time domain. This paper describes a method, called ONE-SHOT for ONe Excitation for Simultaneous High-speed Operation Tomography and a specific hardware implementation of a full FDM-EIT system. A set of mixed sinusoidal voltage excitations is set at all electrodes and the voltage drops across a set of sensing resistors are simultaneously measured. Thereby, the system response is inferred. All projections in the tomographic frame can be performed in a single step, thus enabling the excitation and sensing operation to be continuous. Since digital demodulation with a limited number of samples is considered in our system, restrictions on the choice of AC frequencies are also discussed.

The tomographic method presented in this paper is based on electrical impedance with an array of electrodes. The following sections describe the hardware used involving these four electrodes, with some electromagnetic field simulations followed by a proof-of-concept for a simple 4-electrode system.

## 2. Materials and Methods

### 2.1. Classical Tomography Experiment

#### 2.1.1. Four-Electrodes Tomographic System

Throughout this paper, the principle and operation of the ONE-SHOT is illustrated using a simple EIT system with the number of electrodes $N_{e}=4$. Though this illustrative system used for proof-of-concept consists of relatively smaller number of electrodes than that found in systems currently in use, the main idea is applicable to larger number of electrodes and different data collection strategies with the obvious hardware and software challenges.

**Figure 1:**
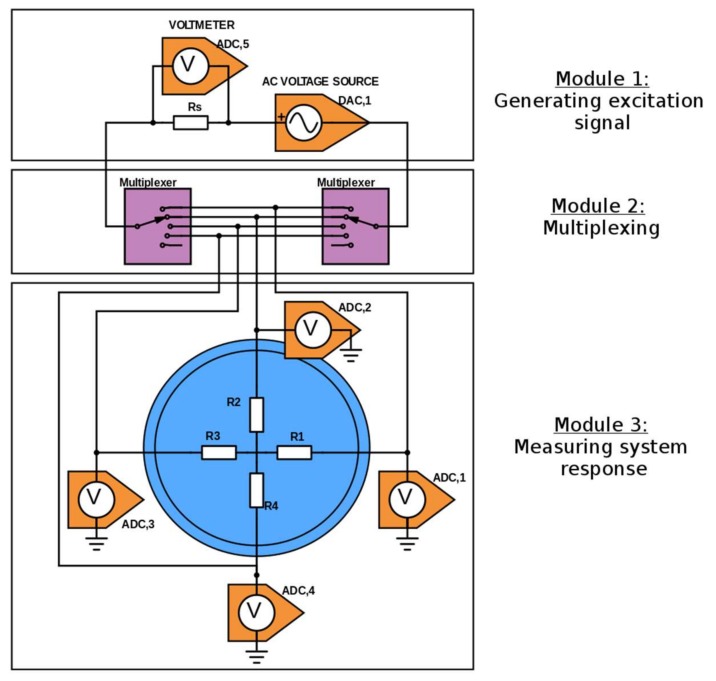
Sketch of the setup for the classical tomography experiment. An AC voltage source generates the excitation signal. Multiplexers route the signal to the target electrodes. Voltmeters measure the system response (voltages). In the blue disk, four resistances represent the mock-up of a test section with the ROI.

Amongst the various data collection strategies (adjacent, opposite, cross, full scan), we consider the full scan strategy and hardware architecture described in [11], illustrated in Figure 1. 

An AC sinewave voltage is generated continuously with a Digital-to-Analog Converter (DAC, 1), in Module 1 of Figure 1. The source and sink of the supply are connected to two multiplexers, shown in Module 2 of Figure 1. The binary address of the selected source and sink electrodes are sent to the multiplexers by digital commands. No electrical current flows through the other electrodes. The electrical current through active electrodes is deduced from the voltage drop measurement (ADC, 5) across a sensing resistor $R_{s}$.

The electrical potential of all electrodes with respect to a common ground is measured with 4 Analog-to-Digital Converters (ADCs 1–4), shown in Module 3 of Figure 1.

#### 2.1.2. Data Collection Strategy

**Figure 2:**
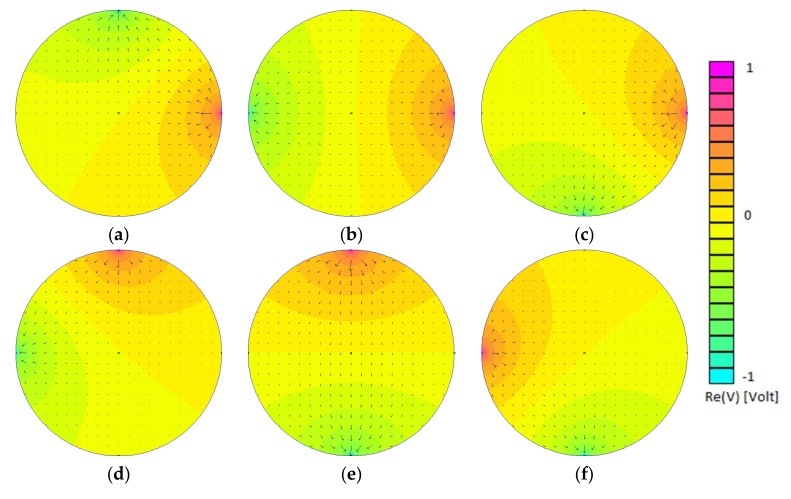
Simulations of the electrical potential and current fields for the 6 excitations performed based on the full scan strategy. Subfigures (**a**–**f**) correspond to 1st, 2nd, 3rd, 4th, 5th and 6th excitations.

Based on the full scan strategy as described in [11], a tomographic sequence consists in sequentially routing the excitation signal to all pairs of electrodes. The projection set includes $N_{p}=6$ excitations with the four-electrode system. The potential and current fields simulated for each excitation with a homogeneous material in the ROI are shown in Figure 2.

Let us define the projection set matrix $P^{SET}$ such as $P_{i,j}^{SET}$ is equal to either +1, −1 or 0 if the *i*th electrode during the *j*th excitation of the set is connected to the source, sink, or ground, respectively:

(1) $$P^{SET}=\left(\begin{array}{rrrrrr} +1 & +1 & +1 & 0 & 0 & 0\\ -1 & 0 & 0 & +1 & +1 & 0\\ 0 & -1 & 0 & -1 & 0 & +1\\ 0 & 0 & -1 & 0 & -1 & -1 \end{array}\right).$$

In other words, each column of the matrix $P^{SET}$ corresponds to the normalized voltages set on each electrode during one excitation.

In the classical approach, one differential AC voltage source is connected sequentially to each pair of electrodes, using mechanical switches or multiplexers. Most systems use AC excitations to avoid polarization effects. The waveform generated from the source can be written as:

(2) $$V_{AC}\left(t\right)=V^{AC}\cdot \sin\left(2\pi f^{AC}t+\phi^{AC}\right),$$

with $f^{AC},V^{AC}\;\mathrm{and}\;\phi^{AC}$ respectively being the frequency, amplitude and phase of the sinusoidal waveform. Please note that $V_{AC}\left(t\right)$ is the sinusoidal waveform while $V^{AC}$ is its amplitude.

One excitation pattern is specified with the waveform $V_{AC}\left(t\right)$ and the index of the pair of electrodes the excitation is routed to. The latter can be expressed as a unit vector $\hat{e_{k}}$ in the base ${ℛ}^{N_{p}}$. Let us refer to the *k*th excitation as:

(3) $$\vec{E_{k}}=V_{AC}\left(t\right)\cdot \hat{e_{k}}.$$

The set of voltages on all electrodes, $\vec{V}$, applied for the *k*th excitation $\vec{E_{k}}$ is:

(4) $$\vec{V}=P^{SET}\cdot \vec{E_{k}}=\left(\begin{array}{rrrrrr} +1 & +1 & +1 & 0 & 0 & 0\\ -1 & 0 & 0 & +1 & +1 & 0\\ 0 & -1 & 0 & -1 & 0 & +1\\ 0 & 0 & -1 & 0 & -1 & -1 \end{array}\right)\cdot \left(\begin{array}{c} \begin{array}{c} 0\\ 0 \end{array}\\ \begin{array}{c} \ldots \\ V_{AC}\left(t\right) \end{array}\\ \begin{array}{c} \ldots \\ 0 \end{array} \end{array}\right).$$

In other words, the standard procedure consists in applying successively the following list of six excitations for the four electrodes involved in the simple system under discussion:

(5) $$\left\{ (\begin{array}{c} V_{AC}\left(t\right)\\ 0\\ 0\\ 0\\ 0\\ 0 \end{array}),(\begin{array}{c} 0\\ V_{AC}\left(t\right)\\ 0\\ 0\\ 0\\ 0 \end{array}),(\begin{array}{c} 0\\ 0\\ V_{AC}\left(t\right)\\ 0\\ 0\\ 0 \end{array}),(\begin{array}{c} 0\\ 0\\ 0\\ V_{AC}\left(t\right)\\ 0\\ 0 \end{array}),(\begin{array}{c} 0\\ 0\\ 0\\ 0\\ V_{AC}\left(t\right)\\ 0 \end{array}),(\begin{array}{c} 0\\ 0\\ 0\\ 0\\ 0\\ V_{AC}\left(t\right) \end{array})\}.\right.$$

The classical paradigm in EIT is to use the same excitation frequency $f^{AC}$ for all excitations in the projection set. Therefore, these excitations are essentially sequential.

### 2.2. The ONE-SHOT Method

The method described in the paper is called ONE-SHOT, as mentioned before, an acronym for ONe Excitation for Simultaneous High-speed Operation Tomography. The main idea consists in selecting a single excitation that is the linear combination of the excitations in the set of Equation (5):

(6) $$\vec{E}=\sum_{k=1}^{N_{p}}\vec{E_{k}}.$$

Firstly, all electrodes will have to be excited with a distinct signal, so the system architecture will have to be modified. Secondly, in order to recover the contribution of each excitation from the measurement of the system response, each excitation $\vec{E_{k}}$ needs to be distinguishable, i.e., having a distinct frequency $f_{k}^{AC}$. Note that in the present work, we have not considered employing different amplitude and phase for each excitation, though this is an obvious continuation of the works related to this method.

In Electrical Impedance Spectroscopy (EIS) or in Multi-Frequency EIT (MF-EIT), there is also the need to combine signal components at different AC frequencies. Yet, the reason for using multiple sinusoidal signals in EIS and MF-EIT is different from that for the ONE-SHOT method. In EIS or MF-EIT, usage of wideband excitation signal avoids the repetition of the same EIT frame acquisition protocol at different AC frequencies [12]. The ONE-SHOT method employs composite of multiple sinusoidal waves in order to reduce the number of steps in the EIT frame acquisition protocol thus making multiplexing superfluous. Research works concerning MF-EIT systems [13,14] describe a digital method to generate composite multi-sine waves with a Field-Programmable Gate Array (FPGA) using the Direct Digital Synthesizer (DDS) technique.

#### 2.2.1. Modified Four-Electrodes System

**Figure 3:**
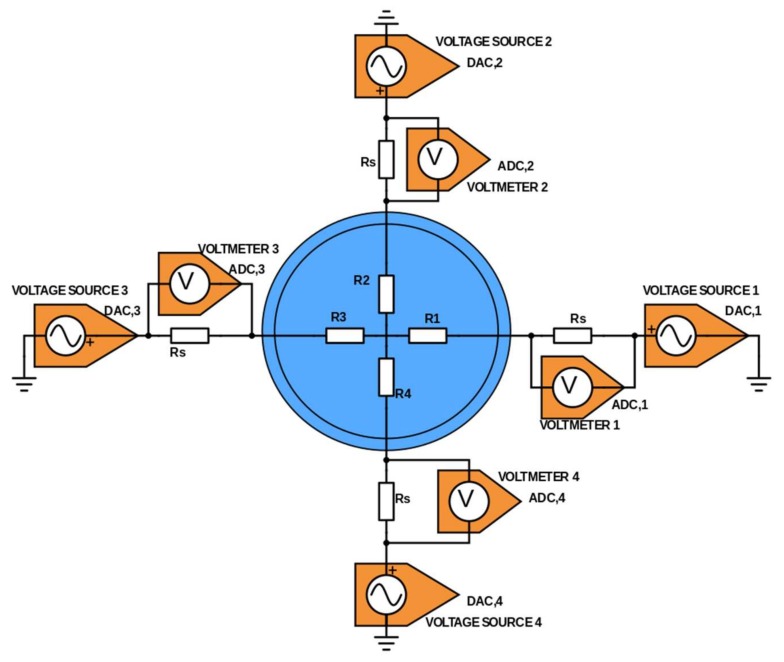
Sketch of the setup for the proof-of-concept experiment. In the blue disk, is the mock-up of a test section. Voltage sources generate the excitation signals, and voltmeters measure the system response (currents) across sense resistors.

The modified tomographic system is schematically presented in Figure 3, obviously with four signal sources generating four different mixed sinusoidal waveforms. The hardware architecture will change from Figure 1 to accommodate these four signal sources which are active continuously, thus making the multiplexers shown in Figure 1 obsolete. The same PXI system (NI PXIe-6368 card, NI PXIe-8840 controller, and NI PXIe-1078 chassis) as in the classical tomography experiment [11] could be used since it features four Digital-to-Analog Converters (DAC) and sixteen Analog-to-Digital Converters (ADC) that can be sampled simultaneously at up to 2 MHz sampling rate. The interfacing circuit for multiplexing had to be redesigned.

In this modified setup, the response of the system is measured with voltage drop measurements across 4 sensing resistors, which provides the electrical current through all electrodes. Each AC voltage source is connected to a single electrode, and the other terminal is connected to a common ground.

This is the reciprocal case of the system in Figure 1, where the electrical potential of the electrodes was measured. There, the AC voltage supply was connected to a pair of electrode, and an additional measurement of the current across a sensing resistor made the system equivalent to systems with a source of current.

#### 2.2.2. Modified Data Collection Strategy

The same set of projections $P^{SET}$ than in Equation (1) is still being considered. Yet, the scan protocol now differs, since a single excitation $\vec{E}$ (Equation (6)) will provide all measurements to deduce the system response:

(7) $$\vec{E}=\left\{\left(\begin{array}{c} \begin{array}{c} V^{AC}\cdot \sin\left(2\pi f_{1}^{AC}t\right)\\ V^{AC}\cdot \sin\left(2\pi f_{2}^{AC}t\right) \end{array}\\ \begin{array}{c} V^{AC}\cdot \sin\left(2\pi f_{3}^{AC}t\right)\\ V^{AC}\cdot \sin\left(2\pi f_{4}^{AC}t\right) \end{array}\\ \begin{array}{c} V^{AC}\cdot \sin\left(2\pi f_{5}^{AC}t\right)\\ V^{AC}\cdot \sin\left(2\pi f_{6}^{AC}t\right) \end{array} \end{array}\right)\right\}.$$

The voltages to be set at each electrode are:

(8) $$\begin{array}{c} \vec{V}=P^{SET}\cdot \vec{E_{k}}=\left(\begin{array}{rrrrrr} +1 & +1 & +1 & 0 & 0 & 0\\ -1 & 0 & 0 & +1 & +1 & 0\\ 0 & -1 & 0 & -1 & 0 & +1\\ 0 & 0 & -1 & 0 & -1 & -1 \end{array}\right)\cdot \left(\begin{array}{c} \begin{array}{c} V^{AC}\cdot \sin\left(2\pi f_{1}^{AC}t\right)\\ V^{AC}\cdot \sin\left(2\pi f_{2}^{AC}t\right) \end{array}\\ \begin{array}{c} V^{AC}\cdot \sin\left(2\pi f_{3}^{AC}t\right)\\ V^{AC}\cdot \sin\left(2\pi f_{4}^{AC}t\right) \end{array}\\ \begin{array}{c} V^{AC}\cdot \sin\left(2\pi f_{5}^{AC}t\right)\\ V^{AC}\cdot \sin\left(2\pi f_{6}^{AC}t\right) \end{array} \end{array}\right)\\ =(\begin{array}{c} V^{AC}\cdot (\sin\left(f_{1}^{AC}t\right)+\sin\left(f_{2}^{AC}t\right)+\sin\left(f_{3}^{AC}t\right))\\ V^{AC}\cdot (\sin\left(f_{1}^{AC}t\right)+\sin\left(f_{4}^{AC}t\right)+\sin\left(f_{5}^{AC}t\right))\\ V^{AC}\cdot (\sin\left(f_{2}^{AC}t\right)-\sin\left(f_{4}^{AC}t\right)+\sin\left(f_{6}^{AC}t\right))\\ V^{AC}\cdot (\sin\left(f_{3}^{AC}t\right)-\sin\left(f_{5}^{AC}t\right)-\sin\left(f_{6}^{AC}t\right)) \end{array}). \end{array}$$

All electrodes need to be excited with a distinct waveform, which explains the need for as many signal generators as number of electrodes in the updated four-electrode system.

#### 2.2.3. ADC and Frequency Selection

The set of waveform generators is implemented with a module of 4 DAC, which can be sampled simultaneously at up to 2 MHz. The measurement of the electrical potentials is performed by a module of 4 ADC, which can be sampled at up to 3 MHz [11].

Implementing the ONE-SHOT method implies constraints on the selection of the frequencies $\left\{f_{i}^{AC},i=1,2,\ldots ,6\right\}$ in order to discriminate the system response to each projection. In this paper, we are using the Discrete Fourier Transform (DFT) of the digital signals as a mean to perform digital demodulation.

Let us consider the sampling frequency $f_{S}$ and the number of samples per channel $N_{S}$. The set of frequencies needs to be extracted from the DFT of the signals. According to the Shannon’s sampling theorem, a signal of frequency f may be recovered if it satisfies the strict inequality:

(9) $$f<\frac{1}{2}f_{S}.$$

For discrete signals with finite number of samples $N_{S}$, this implies that only $N_{f}$ distinct frequencies may be used, with:

(10) $$N_{f}=\frac{N_{S}}{2}\;\mathrm{if}\;N_{S}\;\mathrm{is}\;\mathrm{even},$$

(11) $$N_{f}=\frac{N_{S}+1}{2}\;\mathrm{if}\;N_{S}\;\mathrm{is}\;\mathrm{odd}.$$

In the fastest acquisition mode, the user would select a number of samples that satisfy $N_{f}=N_{p}$, so that enough frequency slots are available for each projection in the set. However, there are some obvious limitations of this mode. Firstly, the first available frequency of the DFT is $f_{0}^{DFT}=0$. Because of the unavoidable DC voltage offsets in any system, DC should be obviously excluded. Secondly, DACs and ADCs introduce non-linearity: they generate harmonic distortion of the input signals. In order to ensure the first order harmonics do not overlap and corrupt signals on other frequency slots of the DFT, one should make sure that the highest frequency $f_{AC,max}$ is less than twice the lowest frequency $f_{AC,min}$ by doubling the number of samples:

(12) $$f_{AC,max}<2\cdot f_{AC,min}.$$

**Figure 4:**
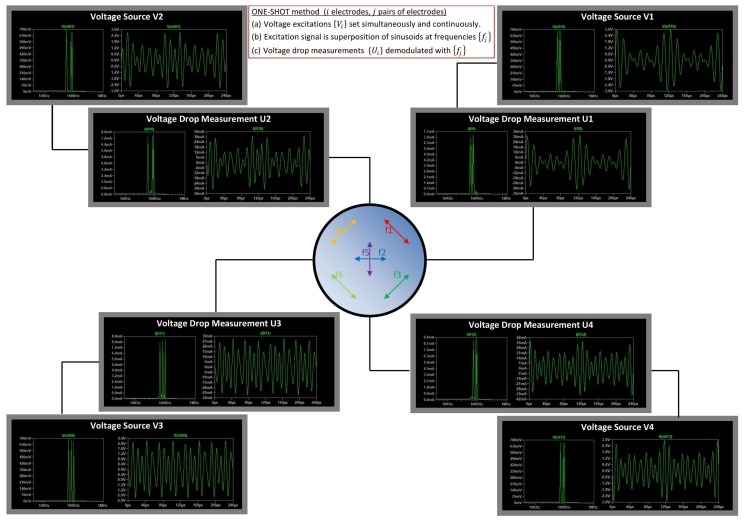
Sketch of the concept of the ONE-SHOT method. Voltage sources generate the excitation signals $\left\{V_{i},i=1,2,3,4\right\}$ as in Equation (8), and electrical currents are deduced from voltage drop measurements $\left\{U_{i},i=1,2,3,4\right\}$. A set of frequencies $\left\{f_{j},j=1,2,3,4,5,6\right\}$ is selected for modulation of mixed sine waves excitation signals and demodulation of measurements.

Based on these two recommendations, the minimum number of samples should satisfy $N_{f}=2\cdot N_{p}+1$. For the four-electrodes system with a set of 6 projections, the number of samples per channel $N_{s}$ is selected such that $N_{s}\geq 25$. The concept of the ONE-SHOT method is illustrated in Figure 4.

### 2.3. Discussion: Pros and Cons

#### 2.3.1. Pros

The main advantage of the method is the absence of transients between successive projections. Indeed, the ONE-SHOT method uses a single and continuous excitation pattern. In general, dead time can be set to accommodate for a transient effect at the expense of the frame acquisition rate. For electrical impedance tomography, this is a great alternative solution to the problem of residual voltages resulting from energy stored in the electrode-electrolyte contact impedance (see [11,15,16]). This challenge in medical applications (skin effect) and flow monitoring (ionic solutions) can be solved by setting a dead time at the detriment of the frame acquisition rate. Other solutions exist to mitigate this parasitic effect such as clamping and discharging or direct coupling circuits, or techniques such as the Over-Zero Switching scheme [4]. Finally, the setup of the system is simplified, since the multiplexing module for routing the excitation signal to target electrodes is obsolete. These electronic components are prone to channel leakage and signal distortion [17].

The second important advantage of the ONE-SHOT method over the standard approach is that at least $N_{P}$ times more samples are available for all measurements since the system response to all projections is measured simultaneously. At a comparable signal-to-noise ratio (SNR) level, a reduction of the deviation due to random noise by a factor of $\sqrt{N_{P}}$ can be expected because of oversampling [18]. The figure of merit is even higher with respect to systems with dead times between projections for signal settling since the ONE-SHOT method employs continuous excitation (there are no transient voltages and no need to implement dead times).

The ONE-SHOT concept can be applied to various tomography techniques based on alternating excitation signals (and variants, e.g., wire-mesh sensor). The assumption is that simultaneously applied excitation signals (at different frequencies) do not interfere.

#### 2.3.2. Cons

The main disadvantage of the ONE-SHOT method is the need for as many sources of excitation as there are electrodes (or transducers) in the system. Another drawback concerns the analysis of the data. The system response is measured at different excitation frequencies for each projection. In some systems, the material properties (attenuation, diffraction, impedance, etc.) might be independent on the frequency of the penetrating wave. In others, the sensitivity of the measurements for a given projection will need to be assessed for the specific frequency associated. Yet, this issue may be mitigated by increasing the number of samples $N_{S}$ and selecting consecutive elements in the DFT for the set of AC frequencies. Indeed, the frequency difference between consecutive elements in the DFT decreases with increasing number of samples:

(13) $$\Delta f=f_{i}^{DFT}-f_{i+1}^{DFT}=\frac{f_{s}}{N_{s}}.$$

## 3. Results

### Proof-of-Concept for 4-Electrode System

A proof-of-concept of the ONE-SHOT method is proposed in the framework of EIT, the research expertise of the authors. The setup, a 4-electrode EIT system and a resistor network to be imaged, has been presented in previous sections and is sketched onto Figure 3. The data collection strategy corresponds to the full scan technique mentioned in previous work [11]. 

The ADCs and DACs are sampled at $f_{s}=200\;\mathrm{kHz}$ synchronously. $N_{s}=128$ samples (per channel) are taken in each tomographic frame. The 13th, 15th, 17th, 19th, 21st and 23rd elements of the DFT have been selected to compose the set of six excitation frequencies $\left\{f_{AC,i}\;i=1,2,\ldots ,6\right\}$. The frequencies are selected so as to facilitate successful interrogation of the media under investigation with enough distance from each other. A mock-up of the ROI of an object to be imaged is built with four resistors $R_{1},R_{2},R_{3},R_{4}$. The value for the sensing resistors for determination of electrical current is selected at 216 Ω. All resistors are measured independently with an Ohmmeter carefully calibrated and tested in the lab. The parameters of the proof-of-concept experiment are given in Table 1.

**Figure 5:**
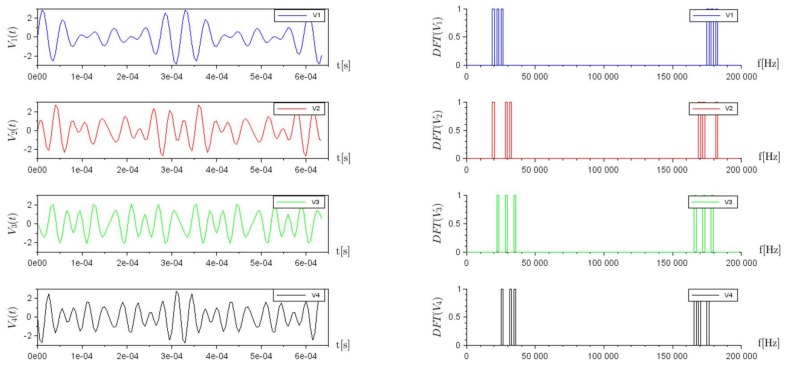
Excitation signals at each of the four electrodes, $V_{1},V_{2},V_{3},V_{4}$. On the left, the time domain representation, and on the right, the frequency domain representation.

The set of voltages $\vec{V}=\left(V_{1},V_{2},V_{3},V_{4}\right)$ on the four electrodes set for the excitation correspond to Equation (8). The time-domain and frequency domain representation of the four signals are shown respectively on the left and right sides of Figure 5.

**Figure 6:**
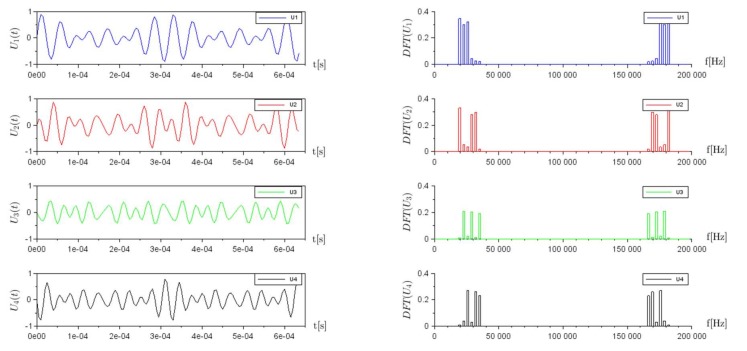
Voltage measurements across the four sensing resistors, $U_{1},U_{2},U_{3},U_{4}$. On the left, the time domain representation, and on the right, the frequency domain representation.

The system response of the mock-up composed of resistors $R_{1},R_{2},R_{3},R_{4}$ is measured as the voltage drop across the four sensing resistors: $U_{1},U_{2},U_{3},U_{4}$. The time-domain and frequency domain representation of the four signals are shown respectively on the left and right sides of Figure 6.

**Figure 7:**
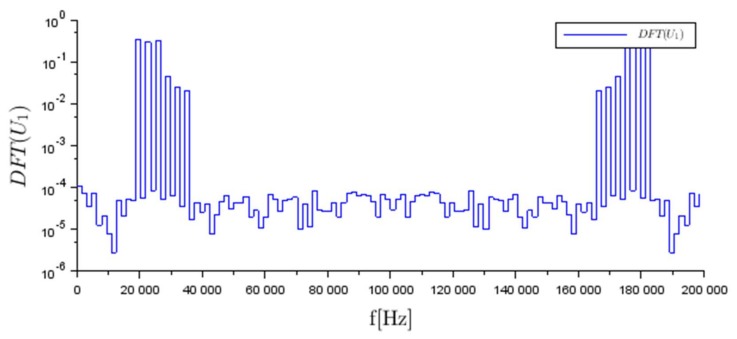
Measurement of the voltage drop $U_{1}$ across the sense resistor for determination of current flow through electrode n°1, in the frequency domain.

The measurements clearly highlight peaks at the six AC frequencies employed for the excitation. The frequency domain representation of $U_{1}$ is repeated onto Figure 7 with a log scale for the y-axis in order to highlight the noise level order of magnitudes below the signal level. The values of SNR for the measurements of $U_{1},U_{2},U_{3}\;\mathrm{and}\;U_{4}$ were 63.8, 64.3, 58.2 and 60.9 dB, respectively. 

The system response of the electrical circuit illustrated onto Figure 3 can easily be modelled. Based on the system response measured experimentally, the reference value of the sensing resistors, and the excitation signal generated, a parametric search of the value of the four resistors $R_{1},R_{2},R_{3},R_{4}$ has been performed. The reference values, the estimates best matching experimental results, and the errors and relative errors of the determination of $R_{1},R_{2},R_{3},R_{4}$ are given in Table 2.

As a conclusion of the experiment, the data obtained with the ONE-SHOT method closely matches the model of the proof-of-concept experiment, indicating there is no major interference between the excitation signals simultaneously applied. No indication of signal distortion has been observed: the DFT elements that do not correspond to the excitation frequencies employed are at least two orders of magnitudes weaker than the DFT elements in the system response.

## 4. Discussion

### 4.1. Conclusions

The main features of the ONE-SHOT method are listed in Table 3 and categorised under the ‘advantage’ or ‘disadvantage’ labels.

The ONE-SHOT method bypasses the classical paradigm of employing the same AC frequency for all excitations. The experiment presented in the paper illustrates some aspects related to multi-frequency excitation, but further efforts are needed to study the performance of ONE-SHOT technique in field tests. 

### 4.2. Future Perspectives

In the current version of the ONE-SHOT method, the mixed excitation signal is composed of the linear combination of single excitations (Equation (6)). Different frequencies have been employed in order to discriminate the system response from the measurements, however, it would also have been possible to use different amplitudes and phases: $V_{k}^{AC}\;\mathrm{and}\;\phi_{k}^{AC}$. The control of these parameters could be used to set excitation signals spanning smaller voltage range, thereby making better use of the full range of DACs. In Figure 4, one can see that the voltages fed to the electrodes 1–4 vary almost from −3 V to 3 V, showing that the current choice for the set of phases $\left\{\phi_{k}^{AC},k=1,2,\ldots ,6\right\}$ is not optimised.

Potential users of the ONE-SHOT method may use a set of frequencies spanning a limited bandwidth, as in [7]. This will mean increasing the number of samples used for the digital demodulation (i.e., a longer integration time). Yet, using an analogy to sliding time windows for time-frequency signal transforms (e.g., Gabor’s transform), it would be possible to maintain a high frame rate. This is a beneficial consequence of using a single and continuous excitation. 

With increasing number of electrodes and subsequent increase in the number of excitation frequencies, the hardware for facilitating ONE-SHOT method may run into crosstalk related problems, unless this issue is properly investigated. Schemes involving matched modulation and demodulation strategies with swept frequency sources and corresponding demodulators at the receiver end with the help of FPGA can help to reduce crosstalk as well as noise related design challenges. Some attempts have been made in biomedical sector using multi-frequency excitation using FPGA for both frequency swept sources and demodulators [19].

In the direction of increased acquisition speed, future works may employ other digital demodulation techniques. In the study of Sun et al. [20], the authors have implemented a digital recursive demodulation method which can analyse signals over less than one period. The authors conclude that the increased acquisition speed comes at the cost of degraded SNR.

## Figures and Tables

**Table 1:** Parameters of the proof-of-concept experiment, signal frequencies and resistors used.

*N_e_*	*N_p_*	*N_s_*	*f_s_* (kHz)	*V^AC^* (volt)	
4	6	128	200	1	
$f_{1}^{AC}$ (kHz)	$f_{2}^{AC}$ (kHz)	$f_{3}^{AC}$ (kHz)	$f_{4}^{AC}$ (kHz)	$f_{5}^{AC}$ (kHz)	$f_{6}^{AC}$ (kHz)
18.750	21.875	25.000	28.125	31.250	34.375
$R_{1}$ (Ω)	$R_{2}$ (Ω)	$R_{3}$ (Ω)	$R_{4}$ (Ω)	$R_{s}$ (Ω)	
383	461	975	674	216	

**Table 2:** Determination of resistors: experimental estimates, reference measurements with Ohmmeter, and errors.

	Reference (Ω)	Estimate (Ω)	Error (Ω)	Relative Error (%)
$R_{s}$	216	--	--	--
$R_{1}$	383	383.96	+0.96	0.25%
$R_{2}$	461	462.18	+1.18	0.26%
$R_{3}$	975	979.25	+4.25	0.44%
$R_{4}$	674	674.82	+0.82	0.12%

**Table 3:** Features of the ONE-SHOT method.

ADVANTAGES	Excitation is continuous: multiplexing is obsolete. Absence of transients: no need for dead time.
	Multiplexers obsolete: no channel leakage.
	Number of samples increases by factor $N_{p}$. Deviations due to random noise are reduced by a factor $\sqrt{N_{p}}$.
	Increased temporal resolution.
	Extension of the method to other modalities.
DISADVANTAGES	$N_{e}$ signal sources required instead of one
	Multi-frequency characterisation of materials required for analysis due to the use of different AC frequencies.
	Additional requirement on the number of samples to avoid overlap of harmonics.

## Supplementary Materials

Supplementary materials can be found at http://www.mdpi.com/1424-8220/18/4/1013/s1. The LabVIEW VI file used in the experiment, the excel spreadsheet with experimental data and analysis and a document concerning the determination of resistor values are published.

## Abbreviations

ADC	Analog-to-Digital Converter
DAC	Digital-to-Analog Converter
DDS	Direct Digital Synthesizer
DFT	Discrete Fourier Transform
EIS	Electrical Impedance Spectroscopy
EIT	Electrical Impedance Tomography
FDM	Frequency-Division Multiplexing
FPGA	Field-Programmable Gate Array
MF-EIT	Multi-Frequency Electrical Impedance Tomography
ONE-SHOT	ONe Excitation for Simultaneous High-speed Operation Tomography
ROI	Region of Interest
SNR	Signal-to-Noise Ratio
$\hat{e_{k}}$	Unit vector in the base
$\vec{E}$	Linear superposition of excitations $\vec{E_{k}}$, *k* = 1, 2, …, 6
$\vec{E_{k}}$	*k*th excitation in the projection set
$\phi^{AC}$	Phase of AC excitation
$f^{AC}$	Frequency of AC excitation
$f_{i}^{AC}$	AC frequency for the *i*th excitation in the projection set
$f_{s}$	Samping frequency (ADC and DAC)
$N_{e}$	Number of electrodes
$N_{f}$	Distinct frequencies usable in the DFT
$N_{p}$	Number of excitations in the projection set
$N_{s}$	Number of samples per channel
$P^{SET}$	Projection set
${ℛ}^{N_{p}}$	Base of independant excitations for a projection set with $N_{p}$ elements
$R_{i}$	Value of the *i*th resistor of the mock-up
$R_{s}$	Sensing resistor(s)
$U_{i}$	Voltage drop measurement across the *i*th sense resistor
$\vec{V}$	Set of excitation voltages set at the electrodes
$V_{AC}$	Waveform of AC excitation signals
$V^{AC}$	Amplitude of AC excitation
$V_{i}$	Excitation voltage set at the *i*th electrode
