# Supplementary material for: A Simultaneous and Continuous Excitation Method for High-Speed Electrical Impedance Tomography with Reduced Transients and Noise Sensitivity
Source: Sensors (Basel). 2018 Mar 28;18(4):1013. doi: 10.3390/s18041013 (PMC5948595; doi:10.3390/s18041013)
Supplement: Supplementary file 1 [file sensors-18-01013-s001.zip › Supplementary-files/Appendix_OneShot_method.pdf]

The system includes 4 voltage sources (DACs), 4 voltage measuring modules (ADCs).

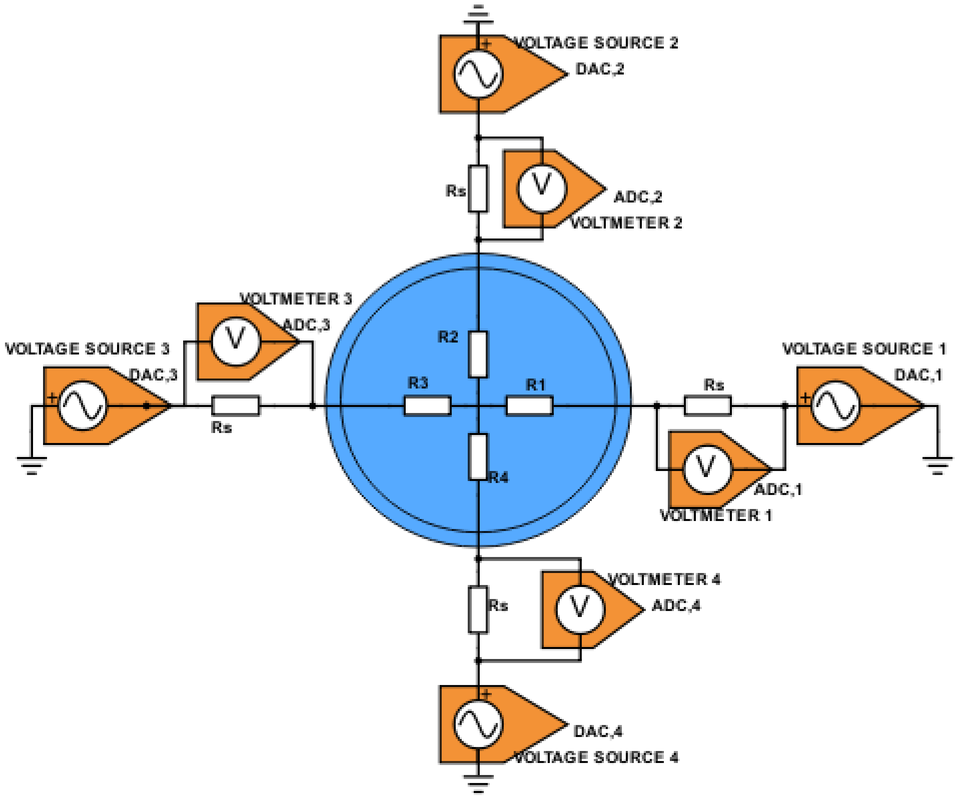

The 4 voltage sources generate the following signals:

$$\begin{aligned} V_1(t) &= +A_1^{\omega_1}\sin(\omega_1 t) + A_1^{\omega_2}\sin(\omega_2 t) + A_1^{\omega_3}\sin(\omega_3 t) \\ V_2(t) &= -A_1^{\omega_1}\sin(\omega_1 t) + A_2^{\omega_4}\sin(\omega_4 t) + A_2^{\omega_5}\sin(\omega_5 t) \\ V_3(t) &= -A_1^{\omega_2}\sin(\omega_2 t) - A_2^{\omega_4}\sin(\omega_4 t) + A_3^{\omega_6}\sin(\omega_6 t) \\ V_4(t) &= -A_1^{\omega_3}\sin(\omega_3 t) - A_2^{\omega_4}\sin(\omega_4 t) - A_3^{\omega_6}\sin(\omega_6 t) \end{aligned} \tag{1}$$

The voltage drop measurements across the sense resistors have 6 frequency components each:

$$\begin{aligned} U_1 &= \sum_{k=0}^{6} U_1^{\omega_k}\sin(\omega_k t) \\ U_2 &= \sum_{k=0}^{6} U_2^{\omega_k}\sin(\omega_k t) \\ U_3 &= \sum_{k=0}^{6} U_3^{\omega_k}\sin(\omega_k t) \\ U_4 &= \sum_{k=0}^{6} U_4^{\omega_k}\sin(\omega_k t) \end{aligned} \tag{2}$$

The (unmeasured) voltage drops U'_1, U'_2, U'_3, U'_4 across the four resistors R_1, R_2, R_3, R_4 respectively, are:

$$U'_1 = \sum_{k=0}^{6} U'^{\omega_k}_1 \sin(\omega_k t) = \sum_{k=0}^{6} R_1.\frac{U_1^{\omega_k}}{R_s}\sin(\omega_k t) \tag{3}$$

$$U'_2 = \sum_{k=0}^{6} U'^{\omega_k}_2 \sin(\omega_k t) = \sum_{k=0}^{6} R_2 . \frac{U_2^{\omega_k}}{R_s} \sin(\omega_k t)$$
$$U'_3 = \sum_{k=0}^{6} U'^{\omega_k}_3 \sin(\omega_k t) = \sum_{k=0}^{6} R_3 . \frac{U_3^{\omega_k}}{R_s} \sin(\omega_k t)$$
$$U'_4 = \sum_{k=0}^{6} U'^{\omega_k}_4 \sin(\omega_k t) = \sum_{k=0}^{6} R_4 . \frac{U_4^{\omega_k}}{R_s} \sin(\omega_k t)$$

Kirchhoff's voltage law relates Equations (1), (2) and (3). Concerning the voltage at frequency ω_1 sources between electrodes 1 and 2, across four resistors (R_1, R_2 and two sense resistors), the constraint writes as:

$$2A_1^{\omega_1} = U_1^{\omega_1} + U_2^{\omega_1} + U'^{\omega_1}_1 + U'^{\omega_1}_2 = U_1^{\omega_1} + U_2^{\omega_1} + (R_1 + R_2)\frac{U_1^{\omega_1}}{R_s} \qquad (4)$$

Leaving all unknown resistor values on the right side of Equation (4) gives:

$$2A_1^{\omega_1} - U_1^{\omega_1} - U_2^{\omega_1} = (R_1 + R_2)\frac{U_1^{\omega_1}}{R_s} \qquad (5)$$

In matrix notation, the problem writes as:

$$\frac{1}{R_s}\begin{pmatrix} U_1^{\omega_1} & U_2^{\omega_1} & U_3^{\omega_1} & U_4^{\omega_1} \\ U_1^{\omega_2} & U_2^{\omega_2} & U_3^{\omega_2} & U_4^{\omega_2} \\ U_1^{\omega_3} & U_2^{\omega_3} & U_3^{\omega_3} & U_4^{\omega_3} \\ U_1^{\omega_4} & U_2^{\omega_4} & U_3^{\omega_4} & U_4^{\omega_4} \\ U_1^{\omega_5} & U_2^{\omega_5} & U_3^{\omega_5} & U_4^{\omega_5} \\ U_1^{\omega_6} & U_2^{\omega_6} & U_3^{\omega_6} & U_4^{\omega_6} \end{pmatrix}\begin{pmatrix} R_1 \\ R_2 \\ R_3 \\ R_4 \end{pmatrix} = \begin{pmatrix} 2A_1^{\omega_1} - U_1^{\omega_1} - U_2^{\omega_1} \\ 2A_1^{\omega_2} - U_1^{\omega_2} - U_3^{\omega_2} \\ 2A_1^{\omega_3} - U_1^{\omega_3} - U_4^{\omega_3} \\ 2A_2^{\omega_4} - U_2^{\omega_4} - U_3^{\omega_4} \\ 2A_2^{\omega_5} - U_2^{\omega_5} - U_4^{\omega_5} \\ 2A_3^{\omega_6} - U_3^{\omega_6} - U_4^{\omega_6} \end{pmatrix} \qquad (6)$$

This problem can be inverted: estimates of $\begin{pmatrix} R_1 \\ R_2 \\ R_3 \\ R_4 \end{pmatrix}$ given a set of measurements and excitations.
